# New Findings on the Survival of *Durusdinium glynnii* Under Different Acclimation Methods to Low Salinities

**DOI:** 10.3390/microorganisms13040946

**Published:** 2025-04-20

**Authors:** Barbara de Cassia S. Brandão, Jéssika L. de Abreu, Deyvid Willame S. Oliveira, Clarissa Vilela F. da Silva Campos, Ingrid M. Thó de Aguiar, Pedro R. de Sena, Alfredo O. Gálvez, Carlos Yure B. Oliveira

**Affiliations:** 1 Laboratory of Live Food Production, Department of Fisheries and Aquaculture, Federal Rural University of Pernambuco, Recife 55810-700, Brazil; soaresbarbara07@hotmail.com (B.d.C.S.B.); jessika.labreu@ufrpe.br (J.L.d.A.); deyvidwillame@gmail.com (D.W.S.O.); clarissavilela19@gmail.com (C.V.F.d.S.C.); ingrid.maguiar@ufrpe.br (I.M.T.d.A.); pedro.rsenar@gmail.com (P.R.d.S.); alfredo_oliv@yahoo.com (A.O.G.); 2 Laboratory of Phycology, Department of Botany, Federal University of Santa Catarina, Florianópolis 88040-535, Brazil

**Keywords:** coccoid, coral symbiosis, mastigote, morphology

## Abstract

This study investigated the effects of salinity on the growth and cell morphotype of the coral-associated dinoflagellate *Durusdinium glynnii* under two acclimation strategies: abrupt saline shock (S5) and gradual reduction (S2). Results revealed optimal growth rates (µ = 0.22–0.35 day^−1^) at salinities of 20–30 g L^−1^, while extreme conditions (10 and 40 g L^−1^) significantly inhibited development. The S2 strategy enabled adaptation to salinities as low as 16 g L^−1^, maintaining higher cell densities compared to the S5 method. Gradual salinity reduction also influenced cellular morphology: below 12 g L^−1^, a predominant shift occurred from motile forms (mastigotes) to non-motile spherical structures (coccoid), suggesting an adaptive response to osmotic stress, gradually reducing the growth rate due to the lower reproductive rate of coccoid cells, as previously reported in studies. The findings conclude that *D. glynnii* is a euryhaline species, tolerant of moderate salinity variations (16–30 g L^−1^) but limited under extreme conditions. Its morphological plasticity and gradual acclimation capacity highlight its potential for cultivation in brackish environments and biomass production for biotechnological applications, such as antioxidants and antimicrobials. The data provide a foundation for future studies on molecular mechanisms of salinity tolerance, essential for coral conservation strategies and bioprospecting efforts.

## 1. Introduction

Microalgae are a diverse group of microorganisms that play a fundamental role in aquatic ecosystems and biogeochemical cycles. Among them, dinoflagellates constitute a lineage characterized by distinctive features, including the arrangement of their flagella, specific pigmentation patterns, specialized organelles, and unique nuclear attributes that differentiate them from other microalgal groups [1]. Dinoflagellates are among the most diverse unicellular microorganisms, playing a crucial role in primary production and global biogeochemical cycles. Within this group, *Durusdinium glynnii*, a member of the family Symbiodiniaceae—formerly classified as *Symbiodinium* clade D—stands out for its mutualistic association with marine invertebrates, particularly reef-building corals [2,3,4,5]. This symbiotic relationship is fundamental for the development and persistence of tropical and subtropical coral reef ecosystems, providing the host with essential nutrients through photosynthesis. Additionally, dinoflagellates contribute to coral health and resilience by enhancing their thermal tolerance and adaptive capacity under environmental stress, which is critical for mitigating the impacts of climate change on reef ecosystems [3,6].

Saline stress is a common factor in natural environments, however, microalgae develop survival strategies to maintain a balance between growth and stress responses, as eukaryotic algae exhibit high plasticity and adaptability to most abiotic stressors [7]. It is also known that the adaptability of microalgae to saline stress varies according to the species’ tolerance level [8]. Coastal environments, being highly dynamic, exhibit abrupt variations in the physicochemical properties of water, with salinity emerging as one of the main stressors for the biological community, influenced by rainfall, tidal cycles, and evaporation [9]. This suggests that *D. glynnii* is naturally subjected to salinity fluctuations in its environment, however, knowledge gaps remain regarding the ecophysiological impacts of this variable on the population of this dinoflagellate species.

Salinity is one of the factors that significantly influence the biology and physiology of dinoflagellates [10]. These organisms, in particular, are capable of crossing the marine/freshwater barrier more frequently than other microalgae, such as chlorophytes and rhodophytes, due to their ability to adapt to new conditions more rapidly and frequently than anticipated [11,12]. In this context, physiological studies emerge as important tools to determine the conditions under which algae thrive. It is known that changes in salinity can alter the dinoflagellate morphology [13,14], potentially slowing cell division, reducing size, disrupting motility, and inducing the formation of palmelloid structures in microalgae. However, some of these changes are species-specific [15], and studies investigating the effects of low salinity concentrations on marine microalgae remain limited in the scientific literature [16].

In summary, understanding how salinity influences the growth and cellular morphology of *D. glynnii* not only enhances our knowledge of its biology but also provides valuable insights for cultivation and biotechnological applications. Optimizing salinity conditions is crucial for laboratory and industrial-scale cultivation, where maintaining stable environments ensures the production of high-quality biomass. From a biotechnological perspective, the ability to cultivate marine algae in lower salinities (5–20 g L^−1^) opens new possibilities for utilizing brackish water—an underexploited resource unsuitable for human consumption or agriculture and costly to desalinate [17]. This approach aligns with several Sustainable Development Goals (SDGs), including SDG 1 (poverty eradication), SDG 8 (economic growth and employment), SDG 10 (reducing inequalities), and SDG 11 (sustainable cities and communities), by fostering economic opportunities in arid, often underdeveloped regions. Additionally, it contributes to SDG 6 (clean water and sanitation) and SDG 14 (life below water) by promoting innovative water use strategies. Moreover, previous studies from our research group have demonstrated that *D. glynnii* is a rich source of bioactive metabolites, including antioxidant [18] and antibacterial [19] compounds, making its biomass a promising candidate for high-value biotechnological applications. Given these prospects, the present study evaluated the impact of salinity on the growth and cellular morphology of *D. glynnii*, comparing two acclimation strategies: abrupt salinity shock and gradual salinity reduction.

## 2. Materials and Methods

### 2.1. Biological Material

The *Durusdinium glynnii* strain (BMK 211) was obtained from the Live Feed Production Laboratory at the Federal Rural University of Pernambuco, where it is maintained in filtered and autoclaved seawater with a salinity of 30 g L^−1^, enriched with f/2 culture medium. The cultures were acclimated to a controlled temperature of 22 ± 1 °C, an irradiance of approximately 150 µmol photons m^−2^ s^−1^, and a 12:12 h light:dark photoperiod.

### 2.2. Experimental Design and Cultivation Conditions

The dinoflagellate pre-culture, initially cultivated in f/2 medium, was transferred to modified Conway medium [20] to avoid nitrogen and phosphorus limitation. Following the transfer, the strain underwent an adaptation period to adjust to the new nutritional conditions, ensuring proper cell development under optimal conditions for the subsequent experiments.

Experiment 1: This study aimed to evaluate the effects of salinity shock stress, exposing the dinoflagellate to salinity levels of 10, 20, 30, and 40 g L^−1^ using sterile seawater (42 g L^−1^, autoclaved at 121 °C for 20 min) and distilled water. In this experiment, the 30 g L^−1^ salinity served as the control treatment. The experimental design was completely randomized, with three replicates per treatment, totaling 12 experimental units over a duration of 18 days. The assay was conducted in 50 mL screw-cap test tubes, which were manually agitated daily to maintain the cells in suspension. The lowest salinity level that maintained satisfactory growth performance was used as the baseline condition for the second phase of the study.

Experiment 2: The objective was to examine the growth capacity of *D. glynnii* under a long-term acclimation strategy. Two strategies for gradual salinity reduction were applied, with intervals of 2 g L^−1^ (S2) and 5 g L^−1^ (S5). The experimental design was also completely randomized, with four replicates per treatment, totaling eight experimental units. The cultures were maintained in borosilicate bottles with a total volume of 1 L and an initial culture volume of 250 mL, under constant aeration (at a rate of 0.05 vvm). Dilutions were performed weekly, whenever the microalgae reached the exponential growth phase, until either a salinity of 0 g L^−1^ was reached or total cell mortality occurred.

Both experiments were conducted under a semi-continuous system with controlled temperature (22 ± 1 °C), irradiance of approximately 150 µmol photons m^−2^ s^−1^, a 12:12 h light:dark photoperiod, and an initial inoculum density of 5 × 10^4^ cells mL^−1^.

### 2.3. Growth Parameters

To evaluate microalgal growth in the conducted studies, 1.5 mL samples were periodically collected from the experimental units and fixed with formaldehyde (4%) for cell quantification using a hemocytometer under a binocular optical microscope. Based on the average density values, logistic growth curves were plotted for each treatment and adjusted according to Pindyck and Rubinfeld [21]. These values were also used to calculate the growth rate (K, divisions day^−1^), doubling time (DT, days division^−1^), and specific growth rate (µ, day^−1^) [22]. For the maximum cell density (MCD, ×10^4^ cells mL^−1^), the highest average cell concentration observed during cultivation was considered, and the day of maximum cell density (dMCD, day) corresponded to the cultivation day on which the MCD was observed.

### 2.4. Cell Morphotype

Weekly, prior to each dilution, 1.5 mL samples were collected from each experimental unit to estimate the number of cells in the motile stage (mastigote) and symbiotic stage (coccoid). For this purpose, two types of sample preparations were performed: unfixed samples, which allowed the counting of coccoid cells present within the hemocytometer grid, and fixed samples, which were used to quantify the total average number of cells. The difference between the quantifications of fixed and unfixed samples corresponded to the density of mastigote cells (Equation (1)).

D*_mast_*_._ = *D_total_* − *D_coc_*_._ (1)

where: D*_mast._* represents the density of cells in the mastigote stage, D*_total_* is the total density of coccoid and mastigote cells, and D*_coc._* represents the density of coccoid cells only.

### 2.5. Statistical Analyses

The results were expressed as mean ± standard deviation. Data were tested for normality and homogeneity using the Shapiro–Wilk and Cochran tests, respectively. Since the data met the assumptions of a normal distribution, they were subjected to analysis of variance (ANOVA), followed by Tukey’s test for mean comparisons. To assess the effect of gradual salinity reduction on the dinoflagellate’s cell density, a quadratic regression model was chosen as it provided the best fit to the data. A significance level of *p* < 0.05 was considered for all analyses.

## 3. Results

### 3.1. Growth Kinetics of Durusdinium glynnii Under Different Salinities

The results of the salinity shock study demonstrate that this variable influences the biological parameters related to the cell production of *Durusdinium glynnii*. At the maximum salinity level tested, the dinoflagellate exhibited the highest division rate (0.42 ± 0.18 div. day^−1^) in the shortest time (2.70 ± 0.96 days div.^−1^), reaching an average density of 30.44 ± 6.34 × 10^4^ cells mL^−1^ at a specific growth rate of 0.62 ± 0.11 day^−1^ on the sixth day of cultivation. Conversely, at the lowest salinity, the dinoflagellate showed a low cell division rate (0.27 ± 0.05 div. day^−1^) and a high doubling time (3.89 ± 0.81 days div.^−1^), achieving a cell density of 27.25 ± 9.28 × 10^4^ cells mL^−1^ at 0.19 ± 0.06 day^−1^ on the eighteenth day of cultivation. The salinity of 20 g L^−1^ did not differ statistically from the control (30 g L^−1^) in any growth parameter. Despite the lower division rate and longer doubling time, both treatments numerically exhibited the highest cell densities, ranging from 35.38 ± 12.79 to 34.38 ± 13.42 × 10^4^ cells mL^−1^ for salinities of 20 and 30 g L^−1^, respectively (Table 1). The trends observed in the growth curves indicate that, at a salinity of 40 g L^−1^, the system reached the stationary phase starting from the 6th day, showing growth inhibition in comparison to the other treatments. The salinity of 10 g L^−1^, in turn, exhibited slower growth, not reaching the stationary phase by the end of the cultivation period. However, it demonstrated a greater growth potential compared to the salinity of 40 g L^−1^, achieving higher cell densities, albeit with a longer growth period. The curve corresponding to the salinity of 20 g L^−1^ was the one that most closely resembled the control treatment. The results indicate that the dinoflagellate *D. glynnii* is capable of adapting to a direct shock with a salinity of 20 g L^−1^ without impairing its growth.

### 3.2. Growth of D. glynnii Under Strategies of Gradual Salinity Reduction

**Figure 1:**
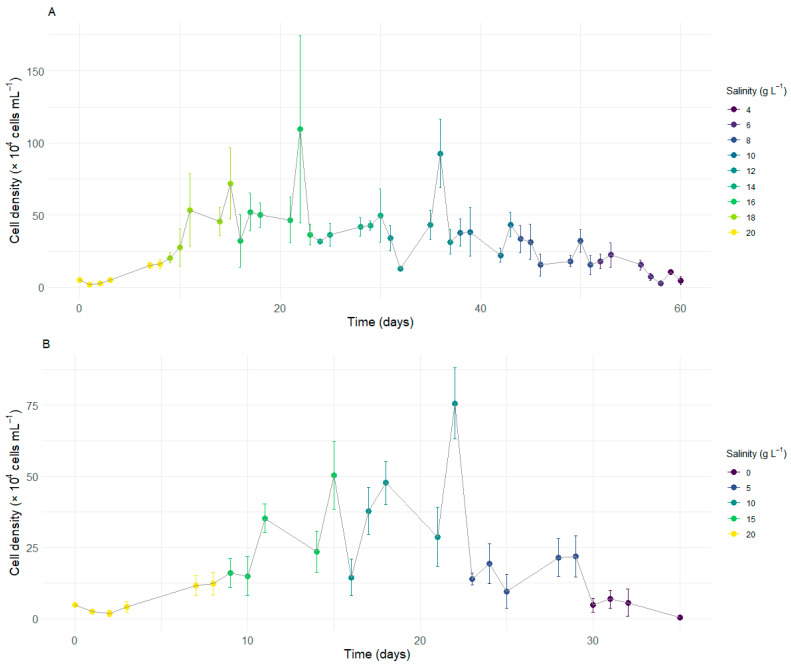
Cell concentration of *Durusdinium glynnii* subjected to two strategies of gradual salinity reduction. Reduction in intervals of S2 (**A**) and S5 (**B**).

Given that *D. glynnii* maintains its growth pattern at a salinity of 20 g L^−1^, the second experiment was initiated under this condition. Strategy S2 exhibited an initial growth trend during the early stages of the experiment, continuing to increase as salinity was gradually reduced to 18 g L^−1^. Upon reduction to a salinity of 16 g L^−1^, a slight decrease in cell density was observed, which remained stable over time, culminating in a peak in density at the end of this period, with an average value of 109.63 × 10^4^ cells mL^−1^. When the salinity was reduced to 14 g L^−1^, a marked decrease in cell density occurred, which later stabilized (Figure 1A).

At a salinity of 12 g L^−1^, a less pronounced initial reduction in density was observed, followed by a significant increase over time, resulting in a second peak at the end of this stage, with an average of 92.75 × 10^4^ cells mL^−1^. Upon reduction to 10 g L^−1^, another sharp decline in cell density was observed, which remained stable until the transition to a salinity of 6 g L^−1^. From this point onward, density began to gradually decrease until reaching a salinity of 2 g L^−1^, where no cells were observed in suspension at the end of the experiment on day 60 (Figure 1A).

Strategy S5 exhibited an initial growth trend during the early stages of cultivation, continuing to increase after the salinity was reduced to 15 g L^−1^. At this stage, the population density rapidly recovered over time, reaching a maximum average value of 50.42 × 10^4^ cells mL^−1^. Upon reducing salinity to 10 g L^−1^, a sharp decline in density was observed. Despite this, *D. glynnii* gradually recovered, eventually reaching the highest average density observed throughout the cultivation for this strategy (75.63 × 10^4^ cells mL^−1^). On the other hand, when salinity was reduced to 5 g L^−1^, a further significant decrease in density occurred, which, in this case, failed to recover over time. Finally, upon reduction to a salinity of 0 g L^−1^, there was a further pronounced decrease in density, culminating in the absence of cells in suspension by the end of the experiment on day 35 (Figure 1B).

**Figure 2:**
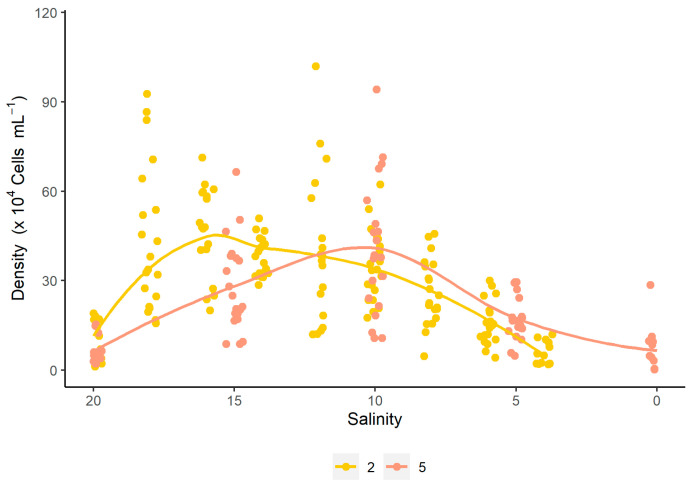
Correlation between cell density and salinity for the different salinity reduction strategies (S2 and S5) for the dinoflagellate *Durusdinium glynnii*.

**Figure 3:**
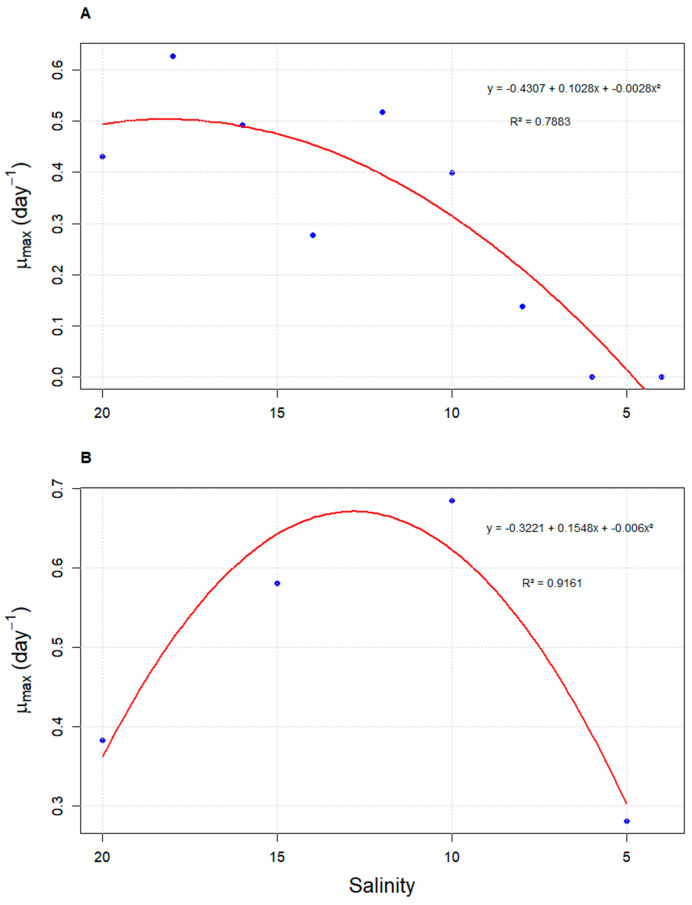
Quadratic regression analysis of the maximum specific growth rate (μ_max_) of *Durusdinium glynnii* cultures as a function of salinity, under two different acclimation strategies to low salinity: S2 (**A**) and S5 (**B**).

The analysis of the density curve as a function of salinity revealed that strategy S2 achieves higher densities in a shorter time compared to strategy S5. However, both curves exhibit an intersection point at a salinity of 12 g L^−1^. Beyond this point, the density in strategy S2 gradually decreases, falling below the values observed for S5. On the other hand, strategy S5 reaches its highest density at a salinity of 10 g L^−1^, before the cell density begins to decrease (Figure 2). These results are consistent with the quadratic regression performed between density and salinity, where the vertex of the parabola passes through the salinity of 12 g L^−1^. Similar to what was observed for cell density, μ_max_ values also varied according to salinity under both evaluated strategies. In strategy S2, the highest values were recorded at higher salinities (18 g L^−1^; μ_max_ = 0.627 ± 0.13 day^−1^), with a sharp decline as salinity decreased. In contrast, strategy S5 showed its peak μ_max_ at 10 g L^−1^ (μ_max_ = 0.685 ± 0.02 day^−1^). Both strategies fitted significantly to quadratic models, with coefficients of determination (R^2^) of 0.7883 and 0.9161 for S2 and S5, respectively (Figure 3).

### 3.3. Effects of Salinite on the Cell Morphotype

**Figure 4:**
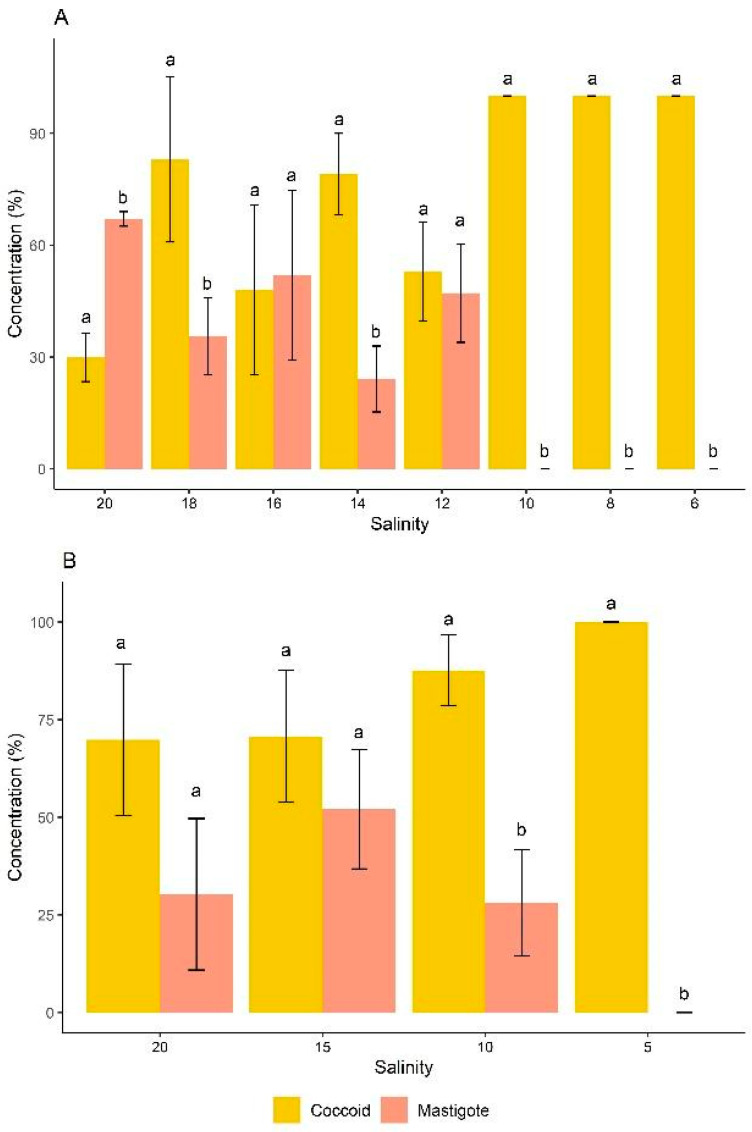
Distribution of cell morphotypes of *D. glynnii* in strategies S2 (**A**) and S5 (**B**) at different salinities. Different letters indicate significant differences according to an ANOVA followed by Tukey’s test (*p* < 0.05).

In the classification of cell morphotypes of *D. glynnii*, strategy S2 exhibited both coccoid cells and mastigotes at salinities ranging from 20 to 12 g L^−1^. Initially, the cells were predominantly mastigotes (approximately 70% of the total population), and as the dilutions progressed, coccoid cells became more prominent. At a salinity of 10 g L^−1^, mastigotes were no longer present, and the population consisted exclusively of coccoid cells, reaching 100% of this morphotype at salinities equal to or below 10 g L^−1^. In strategy S2, all salinities showed statistically significant differences between coccoid and mastigote cell morphotypes, with the exception of salinities 16 and 12 g L^−1^, where no significant differences between these two morphotypes were observed (Figure 4A).

In strategy S5, an inverse pattern was observed, with coccoid cells being dominant throughout the cultivation period. Statistically significant differences between coccoid and mastigote cell morphotypes were observed at salinities 10 and 5 g L^−1^. At salinities 20 and 15 g L^−1^, no significant differences between these two morphotypes were found. Furthermore, at a salinity of 5 g L^−1^, mastigote cells were no longer detected, with 100% of the cell population consisting of coccoid cells (Figure 4B).

## 4. Discussion

Currently, there is limited literature available on isolated cultures of *Durusdinium glynnii*, and our study evaluated, for the first time, the long-term responses of this dinoflagellate to stress caused by salinity variations, expressing its tolerance and survival at low salinity. We observed that, even under stress induced by salinity shock, *D. glynnii* was able to grow in salinities between 10 and 40 g L^−1^, with faster growth rate at 40 g L^−1^ and a longer exponential phase at 10 g L^−1^. A similar behavior was observed for the species *Akashiwo sanguinea*, which exhibited growth within the same salinity range [23]. On the other hand, the species *Alexandrium minutum* exhibited growth between 12 and 37 g L^−1^, with its optimal growth range between 20 and 30 g L^−1^, classifying it as euryhaline [24,25]. Strains of *Ostreopsis* spp. grew within a salinity range of 20 to 40 g L^−1^ [13]. *D. glynnii* exhibited an optimal growth range between salinities of 20 and 30 g L^−1^, resembling *A. minutum*. This plasticity suggests that the dinoflagellate we studied may also be euryhaline, which enables it to adapt to varying salinity conditions.

Despite the reported results, *D. glynnii* showed a clear preference for higher salinity conditions, as evidenced by the reduction in growth rate at lower salinities. At high salinities, it is known that enzymes can be deactivated, leading to a reduction in the photosynthetic rate and consequent loss of cellular water, factors that contribute to decreased cell density [26]. This mechanism explains the short exponential phase observed at a salinity of 40 g L^−1^, with growth inhibition from the sixth day of cultivation, even with a relatively high specific growth rate (0.62 ± 0.11 day^−1^). On the other hand, exposure to low salinities is often associated with oxidative stress in dinoflagellates [27], which may have contributed to the slower adaptation and reduced densities observed in our study at a salinity of 10 g L^−1^ (0.27 ± 0.05 div. day^−1^). *A. minutum* exhibited similar behavior to *D. glynnii* when cultivated at 10 g L^−1^, with very slow growth (~0.15 divisions day^−1^) and higher growth rates at elevated salinities [24].

Quantifying the growth rate provides valuable information regarding overall health and response to specific conditions, as it allows for the assessment of the organism is ability to adapt to different environments [28]. Within the optimal growth range (20 and 30 g L^−1^), the specific growth rate (µ) of *D. glynnii* ranged from 0.22 to 0.35 day^−1^. The dinoflagellate *Alexandrium tamiyavanichii*, which also grew well between 20 and 30 g L^−1^ (µ = 0.25 to 0.35 day^−1^) [25], exhibited similar results. For *Vulcanodinium rugosum*, the salinities that favored these results were 25 and 40 g L^−1^ (µ = 0.21–0.39 day^−1^) [10]. *Prorocentrum micans* showed better growth at a salinity of 30 g L^−1^ (µ = 0.38 day^−1^) and low growth at 25 g L^−1^ (µ = 0.15 day^−1^), while *P. obtusum* exhibited similar growth [27]. This comparison highlights that, although these dinoflagellate species share similar specific growth rates, their salinity preferences vary, reflecting species-specific adaptations.

During the salinity shock experiment, *D. glynnii* reached a maximum cell density of 35.38 ± 12.79 × 10^4^ cells mL^−1^. Previous studies reported that this same species reached a maximum density of 70.25 ± 2 × 10^4^ and 91.83 ± 16.83 × 10^4^ cells mL^−1^ [28,29]. A reasonable explanation for the lower density observed in the present study is that the salinity shock experiment was conducted in test tubes without aeration, reducing the oxygen concentration in the culture medium, which likely favored slower growth or lower cell density. Regarding gradual acclimation, the highest cell density in strategy S2 was observed at a salinity of 16 g L^−1^, while in strategy S5, it occurred at a salinity of 10 g L^−1^. Although these salinities are similar, S2 achieved approximately 32% higher cell density compared to S5, suggesting that more gradual salinity reductions are more effective in promoting biomass production.

During the gradual acclimation process, it was observed that, starting at a salinity of 6 in strategy S2 and at a salinity of 5 g L^−1^ in strategy S5, there was a more pronounced decline in the growth curve. Under these conditions, the species was no longer able to adapt, indicating that this may be the lethal salinity threshold for *D. glynnii*. This behavior may be related to the osmoregulation process, which requires high energy expenditure from the cells, compromising growth rates [29,30]. Another important factor to consider is the age of the cultures, as the gradual salinity reduction test in the present study lasted between 35 and 60 days. Aged cultures of dinoflagellates show a decrease in the production of genes related to stress/stimulus responses when exposed to abiotic stress conditions, resulting in increased cell mortality in response to external stress in marine microalgal populations with the advancing chronological age of the culture [31,32,33]. Based on this, we hypothesize that the aging of *D. glynnii* cultures, combined with extreme salinity reduction, reduces cell motility, compromises stress responses, and increases cell mortality. This suggests that cells progressively become less adaptable and more vulnerable as they age, which may affect the survival of the dinoflagellate.

Most symbiotic species have the potential to transition between a free-living motile stage (mastigote) and a spherical symbiotic stage (coccoid) [34]. Microalgae respond to abiotic stress through various mechanisms, with morphological changes such as cell size alterations, flagellar loss, and reduction of motile cells as culture age increases [35,36]. This pattern was corroborated by the present study, which observed both cell morphotypes, with coccoid cells being predominant. This predominance progressively increased, reaching 100% coccoid cells as the stress caused by decreasing salinity intensified, demonstrating a clear transition from mastigote to coccoid cells in both strategies S2 and S5. A previous study indicated that under optimal growth conditions, *D. glynnii* was composed predominantly of mastigote cells [30]. The same authors further reported that thermal stress reduced this percentage to below 90%, except when there was a high urea concentration, which favored mastigotes (>95%). Conversely, low nitrate concentrations induced a predominance of coccoid cells during thermal stress. Despite the application of different types of stress, both studies indicate a transition between morphotypes, with environmental stress favoring the formation of coccoid cells, but suggesting that the factors inducing this transition vary depending on the type of stress applied.

## 5. Conclusions

Salinity has been shown to be a key variable for the growth of the dinoflagellate *Durusdinium glynn*ii, with an optimal range between 20 and 40 g L^−1^. The experimental results suggest that this species can survive and maintain competitiveness in brackish water habitats, with the potential for adaptation to salinities up to 16 g L^−1^ through strategy S2, without significantly compromising cellular growth. Although it does not guarantee maximum performance under low salinity conditions, cultivation under these conditions holds potential for optimizing the production of biocompounds of biotechnological interest. These findings provide fundamental insights into the ecophysiological behavior of *D. glynnii* in response to salinity variations, an area still underexplored in the scientific literature. This preliminary study establishes basic parameters for the growth and adaptation of the species, which may serve as a foundation for future, more in-depth investigations. Subsequent research could explore the molecular, biochemical, and metabolic mechanisms underlying the observed plasticity, thus broadening the potential applications of this dinoflagellate in biotechnology and ecological applications.

## Figures and Tables

**Table 1:** Growth parameters of the dinoflagellate *Durusdinium glynnii* subjected to stress from different salinities.

Parameters	Salinity (g L^−1^)			
	10	20	30	40
K (divisions day^−1^)	0.27 ± 0.05 ^a^	0.23 ± 0.02 ^a^	0.23 ± 0.05 ^a^	0.42 ± 0.18 ^a^
DT (days division^−1^)	3.89 ± 0.81 ^ab^	4.32 ± 0.32 ^ab^	4.42 ± 0.88 ^a^	2.70 ± 0.96 ^b^
µ (day^−1^)	0.19 ± 0.06 ^c^	0.35 ± 0.03 ^b^	0.22 ± 0.03 ^bc^	0.62 ± 0.11 ^a^
MCD (×10^4^ cells mL^−1^)	27.25 ± 9.28 ^a^	35.38 ± 12.79 ^a^	34.38 ± 13.42 ^a^	30.44 ± 6.34 ^a^
dMCD (day)	18	6	6	6

Mean ± standard deviation (*n* = 3). Mean values within the same row with different letters indicate significant differences according to an ANOVA followed by Tukey’s test (*p* < 0.05). K, growth rate; DT, doubling time; µ, specific growth rate; MCD, maximum cell density; dMCD, day of maximum cell density.

## Data Availability

The original contributions presented in this study are included in the article. Further inquiries can be directed to the corresponding author.
